# Urinary proteomics of Henoch-Schönlein purpura nephritis in children using liquid chromatography-tandem mass spectrometry

**DOI:** 10.1186/s12014-020-09274-x

**Published:** 2020-03-12

**Authors:** Xiang Fang, Heyan Wu, Mei Lu, Yan Cao, Ren Wang, Meiqiu Wang, Chunlin Gao, Zhengkun Xia

**Affiliations:** 1 Department of Pediatrics, Jinling Hospital, the First School of Clinical Medicine, Southern Medical University, No. 305 Zhongshan East Road, Nanjing, 210002 Jiangsu China; 2 Department of Clinical Medicine, Anqing Medical College, Anqing, 246052 Anhui China; 3 Nanjing Maternity and Child Health Care Institute, Women’s Hospital of Nanjing Medical University, Nanjing Maternity and Child Health Care Hospital, Nanjing, 210004 Jiangsu China

**Keywords:** Henoch-Schönlein purpura nephritis, Urine, Proteomics, Children, Mass spectrometry

## Abstract

**Background:**

Henoch-Schönlein purpura nephritis (HSPN) is the principal cause of morbidity and mortality in children with Henoch-Schönlein purpura (HSP). However, the criteria for risk assessment currently used is not satisfactory. The urine proteome may provide important clues to indicate the development of HSPN.

**Methods:**

Here, we detected and compared the urine proteome of patients with HSPN and healthy controls by liquid chromatography-tandem mass spectrometry (LC–MS/MS) in the data-independent acquisition (DIA) mode. The differentially expressed proteins were analysed by gene ontology (GO) analysis and Kyoto Encyclopedia of Genes and Genomes (KEGG) analysis. For validation, enzyme-linked immunosorbent assay (ELISA) was used to analyse the selected proteins.

**Results:**

A total of 125 proteins (29 upregulated and 96 downregulated) were found to be differentially expressed in children with HSPN compared with the controls. Forty-one proteins were predicted to have direct interactions. The enriched pathways mainly included focal adhesion, cell adhesion molecules, the PI3K-Akt signalling pathway, ECM-receptor interactions and so on. Cell adhesion related to the pathogenesis of HSPN was the main biological process identified in this study. The decrease in two proteins (integrin beta-1 and tenascin) was validated by ELISA.

**Conclusions:**

Our study provides new insights into the assessment of HSPN progression in children, as well as new potential biomarkers. The data confirm the value of the urinary proteome in capturing the emergence of HSPN.

## Background

Henoch-Schönlein purpura (HSP) is the most common systemic vasculitis involving capillaries and the deposition of immunoglobulin A immune complexes in childhood. The annual incidence of HSP varies between 10 and 30 cases per 100 000 children < 17 years old, and most cases occur in children < 10 years of age [1]. Up to half of children with HSP develop Henoch-Schönlein purpura nephritis (HSPN), 90% of which occurs within 6 months of onset [2, 3]. Most patients are diagnosed with HSPN at grade III pathology [4]. Other organ manifestations of HSP are mostly benign and self-limiting, but HSPN may lead to chronic renal disease and end-stage renal failure [5]. Thus, renal involvement may be the principal cause of morbidity and mortality in children with HSP. It is important to clarify the onset mechanism and perform early diagnosis to improve the treatment and prognosis of HSPN. Renal biopsy is the gold standard for detecting renal changes, but it is an invasive examination that cannot be tolerated by most people, and its risks involved do not permit repeated inspection.

In recent years, urine has been considered a potential source to provide important clues regarding the progression of diseases [6]. The normal excretion of proteins in urine is less than 150 mg/L per 24 h, and approximately 30% of urinary protein originates from plasma via blood filtration, whereas the remaining 70% is derived from the kidneys. In patients with HSPN, the filtration barrier is injured, leading to a massive loss of proteins in urine (proteinuria). Urinary proteomics as a novel tool has become an efficient approach for predicting renal involvement prior to renal biopsy and biomarker discovery in kidney diseases [7]. Liquid chromatography-tandem mass spectrometry (LC–MS/MS) is a noninvasive detection method used in urine proteomics that can provide qualitative and quantitative assessments of the proteome and offer clues for biological processes in systems. LC–MS/MS has been applied in the proteomic analysis of various kidney diseases, such as membranous nephropathy [8], acute kidney injury [9], lupus nephritis [10] and IgA nephropathy (IgAN) [11]. Data-independent acquisition (DIA) by LC‑MS/MS, which enables comprehensive mapping of the urinary proteome, has sufficient throughput to perform biomarker discovery studies, as all identified proteins can be precisely quantified in hundreds of samples [12].

However, there are few reports of urinary protein alterations focusing on the risk of childhood HSPN. HSPN is characterized by mesangial IgA deposits that induce mesangial cell proliferation and extracellular matrix expansion [13, 14], which are the most common and major pathological change of paediatric HSPN. Some children even have segmental glomerulosclerosis or crescent formation with a loss of functional nephrons in progressive HSPN [13]. The process invariably ends with global glomerular sclerosis under the action of inflammatory mediators. We believe that the roles played by the proteins are worth discussing. In the current study, we aimed to identify the biological significance of differentially expressed proteins from paediatric patients with HSPN via LC‑MS/MS in the DIA mode. We expected that some of the differentially expressed proteins could indicate the biological processes involved in the development of HSPN and that the proteomic profile might provide some hint for the treatment and prognosis of HSPN in children.

## Materials and methods

### Patients and urine samples

Midstream morning urine samples were collected from patients with biopsy-proven HSPN (Group A, n = 34) visiting the Department of Pediatrics, Jinling Hospital, Nanjing, China, from July 2018 to October 2019 (Additional file 1). The enrolment criteria were as follows: (1) age from 2 to 16 years; (2) abnormal urinalysis, including hematuria and/or proteinuria, within 6 months of HSP or renal biopsy results of mesangial proliferative glomerulonephritis characterized by an IgA mesangial deposition [15]; and (3) cases with congenital or hereditary diseases and with severe cardiopulmonary diseases were excluded from the present study. Early-morning spot urine from healthy volunteers (Group B, n = 33) and HSP children (Group C, n = 10) was also collected (Additional files 2 and 3). Since HSPN originates from HSP in children, urine samples from HSP children were collected for further validation to exclude possible false positives. The baseline characteristics of the participants are shown in Table 1. Among them, urine samples from group A and group B (4 children in each group) were detected using the DIA method via LC‑MS/MS. Thirty samples from group A, 29 from group B and 10 from group C were incorporated into the validation cohort. Samples were kept at room temperature for less than 4 h, followed by low-speed centrifugation at 2000*g* for 10 min at room temperature to remove cellular debris and were then stored at − 80 °C until use. The study was approved by the Ethics Committee of Jinling Hospital (2019nzgkj-159).

**Table 1:** Baseline characteristics of patients for validation

Characteristics	HSPN (n = 30)	Normal (n = 29)	HSP (n = 10)
Onset age (years)	9.4 ± 2.4	8.8 ± 2.9	9.3 ± 2.5
Male gender n (%)	20(66.7%)	12(41.4%)	6(60%)
Presenting symptoms n (%)			
Macro or microscopic hematuria	30(100%)	–	–
Proteinuria	27(90%)	–	–
Purpuric rash	30(100%)	–	10 (100%)
Colicky pain	13(43.3%)	–	6(60%)
Arthralgia	11(36.7%)	–	5(50%)
Edema	–	–	–
Bloody stools	–	–	–
Renal pathology n (%)			
IgA mesangial deposition	30(100%)	–	–
Mesangial proliferation	30(100%)	–	–
Crescents	15(50%)	–	–
Segmental glomerulosclerosis	13(43.3%)	–	–
Laboratory measurements			
Serum IgA levels (g/l)	1.8 ± 0.5	–	1.9 ± 0.3
Serum IgE levels (IU/ml)	193.3 ± 182.9	–	285.3 ± 106.7
Serum creatinine (μmol/l)	36.0 ± 8.5	–	34.0 ± 5.4
eGFR (ml/min/1.73 m^2^)	200.7 ± 31.3	–	203.8 ± 15.3

eGFR is estimated by the Schwartz formula [K* height (cm)/serum creatinine (μmol/L)]

*HSPN* Henoch-Schönlein purpura nephritis, *Normal* healthy controls, *HSP* Henoch-Schönlein purpura, *eGFR* estimated glomerular filtration rate

### Protein preparation and digestion

Equal volume samples were concentrated to the same volume by ultrafiltration. Lysis buffer (4% SDS, 100 mM DTT, 150 mM Tris–HCl pH 8.0) was added to the samples directly. The lysates were boiled for 15 min using a homogenizer (Fastprep^®^-24, MP Biomedical, USA) and broken by ultrasound for 3 min. After centrifugation at 14,000*g* for 40 min, the supernatant was quantified using the BCA Protein Assay Kit (Bio-Rad, USA).

Protein digestion was performed according to the FASP procedure described by Wisniewski [16]. Briefly, 200 μg of protein was incorporated into 30 μl SDT buffer (4% SDS, 100 mM DTT, 150 mM Tris–HCl pH 8.0). The detergent, DTT and other low molecular weight components were removed using UA buffer (8 M urea, 150 mM Tris HCl pH 8.0) by repeated ultrafiltration (Microcon units, 30 kD, Millipore, USA). One hundred microliters of 0.05 M iodoacetamide in UA buffer was added to block reduced cysteine residues. Then, the samples were incubated for 30 min in the dark. The filters were washed with 100 μl UA buffer three times and then 100 μl 25 mM NH_4_HCO_3_ twice. Finally, the protein suspensions were digested with 2 μg trypsin (Promega, USA) in 40 µl 100 mM NH_4_HCO_3_ buffer overnight at 37 °C. The resulting peptides were collected as a filtrate. The peptide content was estimated by the spectral density of UV light at 280 nm.

Digested pool peptides were then fractionated into 10 fractions using a Thermo Scientific™ Pierce™ High pH Reversed-Phase Peptide Fractionation Kit. Each fraction was concentrated by vacuum centrifugation and reconstituted in 15 µl of 0.1% (v/v) formic acid. Collected peptides were desalted on C18 Cartridges [Empore™ SPE Cartridges C18 (standard density), bed I.D. 7 mm, volume 3 ml, Sigma]. HRM calibration peptides (Biognosys) were added to the samples before analysis according to the manufacturer’s instructions for the DIA experiments.

### LC‑MS/MS by Q Exactive HF

MS experiments were performed on a Q Exactive mass spectrometer that was coupled to an Easy nLC 1200 chromatography system (Thermo Scientific, Waltham, MA, USA). Each DIA cycle contained one full MS-SIM scan, and 30 DIA scans covered a mass range from 350 to 1650 m/z with the following settings: the SIM full scan resolution was 60,000 at 200 m/z; AGC, 3e6; maximum IT, 50 ms; and profile mode; the DIA scans were performed at a resolution of 30,000; AGC target, 3e6; Max IT, auto; and normalized collision energy, 30 eV. Peptides (2 μl of digest) were separated by a linear gradient from buffer B (80% acetonitrile and 0.1% formic acid) at a flow rate of 250 nl/min. The total run time with the loading and washing steps was 120 min. The column oven was set to 40 °C.

### Sequence database search and data analysis

The MS data were analysed using MaxQuant software version 1.5.3.17. The database was downloaded from the website http://www.uniprot.org. iRT peptide sequences were added (> Biognosys|iRT Kit|Sequence_fusion: LGGNEQVTRYILAGVENSKGTFIIDPGGVIRGTFIIDPAAVIRGAGSSEPVTGLDAKTPVISGGPYEYRVEATFGVDESNAKTPVITGAPYEYRDGLDAASYYAPVRADVTPADFSEWSKLFLQFGAQGSPFLK). The parameters were set as follows: the enzyme was trypsin, maximum missing cleavage was 2, fixed modification was carbamidomethyl (C), and dynamic modification was oxidation (M) and acetyl (Protein N term). All the reported data were based on the 99% confidence interval for protein identification as determined by the false discovery rate (FDR = N (decoy)*2/(N(decoy) + N(target))) ≤ 1%. A spectral library was constructed by importing the original raw files. DIA data were analysed with a Spectronaut Pulsar X™ search of the above constructed spectral library. All the results were filtered based on a Q value cutoff of 0.01 (equivalent to FDR < 1%).

Protein intensity was calculated by summing the peptide peak areas of each protein from the Spectronaut output file. To test the spectral libraries, one urinary sample was measured three times using the Q Exactive HF and was then analysed in Spectronaut. The results were benchmarked based on the numbers of detected peptides and proteins as well as the reproducibility of the peptide and protein detection. For the statistical and bioinformatic analysis, only the urinary proteins present in at least 3 patients in the same group were used.

### Bioinformatics analyses

The proteins were defined as differentially expressed if the fold-change of comparing the disease groups and healthy controls was ≥ 2 or ≤ 0.5 and the *P* value < 0.05. The differentially expressed proteins were analysed by hierarchical clustering to classify all samples (http://en.wikipedia.org/wiki/Cluster_analysis). Next, the differentially expressed proteins were subjected to gene ontology (GO) analysis by Blast2GO (https://www.blast2go.com/) and matched against the Kyoto Encyclopedia of Genes and Genomes (KEGG) database by the KEGG Automatic Annotation Server (KAAS, https://www.genome.jp/tools/kaas/). P < 0.05 in Fisher’s exact test was considered significant. Protein‑protein interaction (PPI) networks were created for these proteins using the STRING database (http://string-db.org/).

### Validation by ELISA analysis

All urinary samples were thawed, centrifuged and aliquoted according to the manufacturer’s instructions for ELISA kits (CUSABIO Biotech CO., LTD). Then, 100 μl of a biotin-conjugated antibody (1:100 dilution) and 100 μl of horseradish peroxidase (HRP)-avidin (1:100 dilution) were added to 100 μl of each sample after centrifugation at 10,000*g* for 15 min. After development with the TMB substrate solution, the reaction was terminated by adding 50 μl of the stop solution. The optical density (OD) values were read at 450 nm, and the concentrations were automatically calculated according to the standard curve.

### Statistical analysis

We used SPSS software version 19.0 for Windows (IBM, Chicago, IL, USA) for statistical analyses. The data were subjected to the Shapiro–Wilk normality test. Continuous variables are expressed as means ± standard deviations. Categorical variables are expressed as absolute numbers and percentages (%). The significance of the protein abundance changes was calculated using nonparametric Student’s t test, and Bonferroni multiple testing correction was applied. A two-tailed test with p < 0.05 was considered significant. Graphs were prepared using GraphPad Prism version 7.0 (GraphPad Software, San Diego, CA, USA).

## Results

### Integrated proteome information

**Fig. 1:**
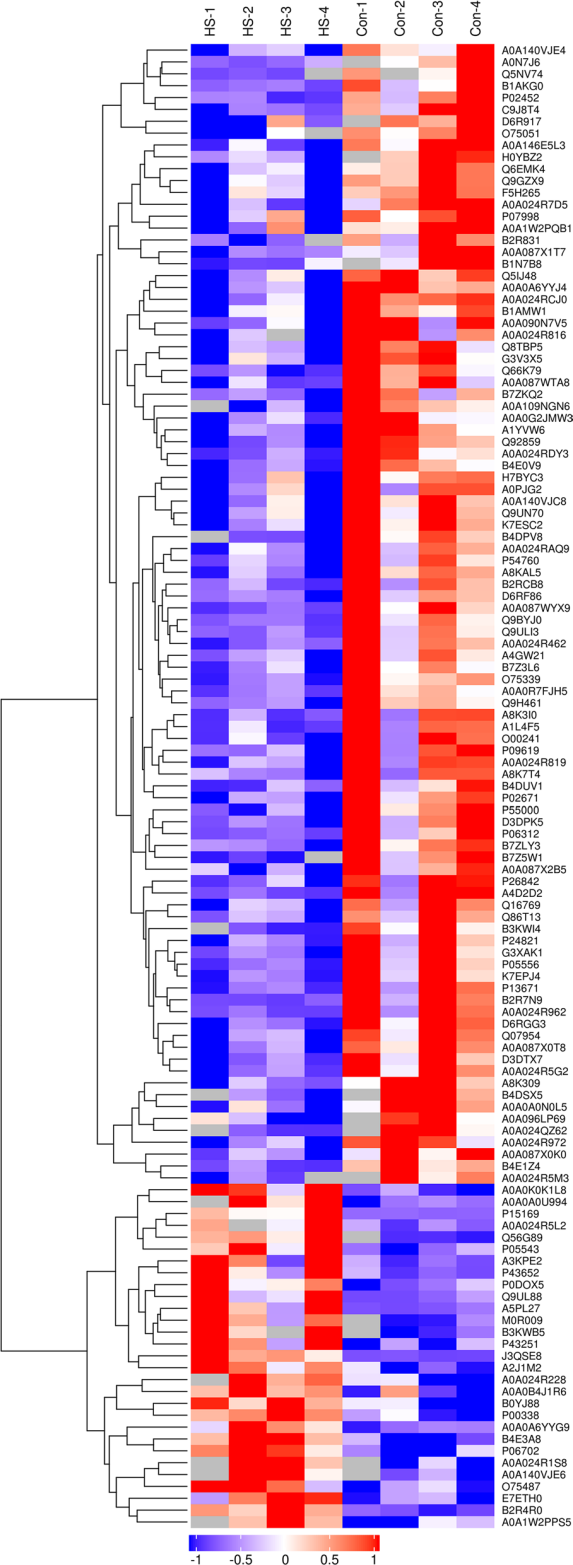
Cluster analysis of 125 differentially expressed proteins. The hierarchical clustering results are represented as a tree heat map, with the ordinate representing significantly differentially expressed proteins and the abscissa representing sample information. Significant differences in protein expression in the different numerical expression quantities (log2 expression) of the samples with different colours are shown in the heat map, where red represents significantly upregulated proteins, green represents significantly downregulated proteins, and grey represents no quantitative information for proteins. HS-1, 2, 3, and 4 represent patients with Henoch-Schönlein purpura nephritis, and Con-1, 2, 3, and 4 represent the healthy control group

A total of 1209 non‑redundant proteins were detected in urine from HSPN patients, and 125 proteins (29 upregulated and 96 downregulated) were found to be differentially expressed in HSPN patients compared with those from healthy controls (Additional file 4). Hierarchical clustering analysis was conducted on 125 dysregulated proteins, and the heatmap obtained from the analysis provided protein profiles across the HSPN group and healthy controls (Fig. 1).

### GO functional analysis

**Fig. 2:**
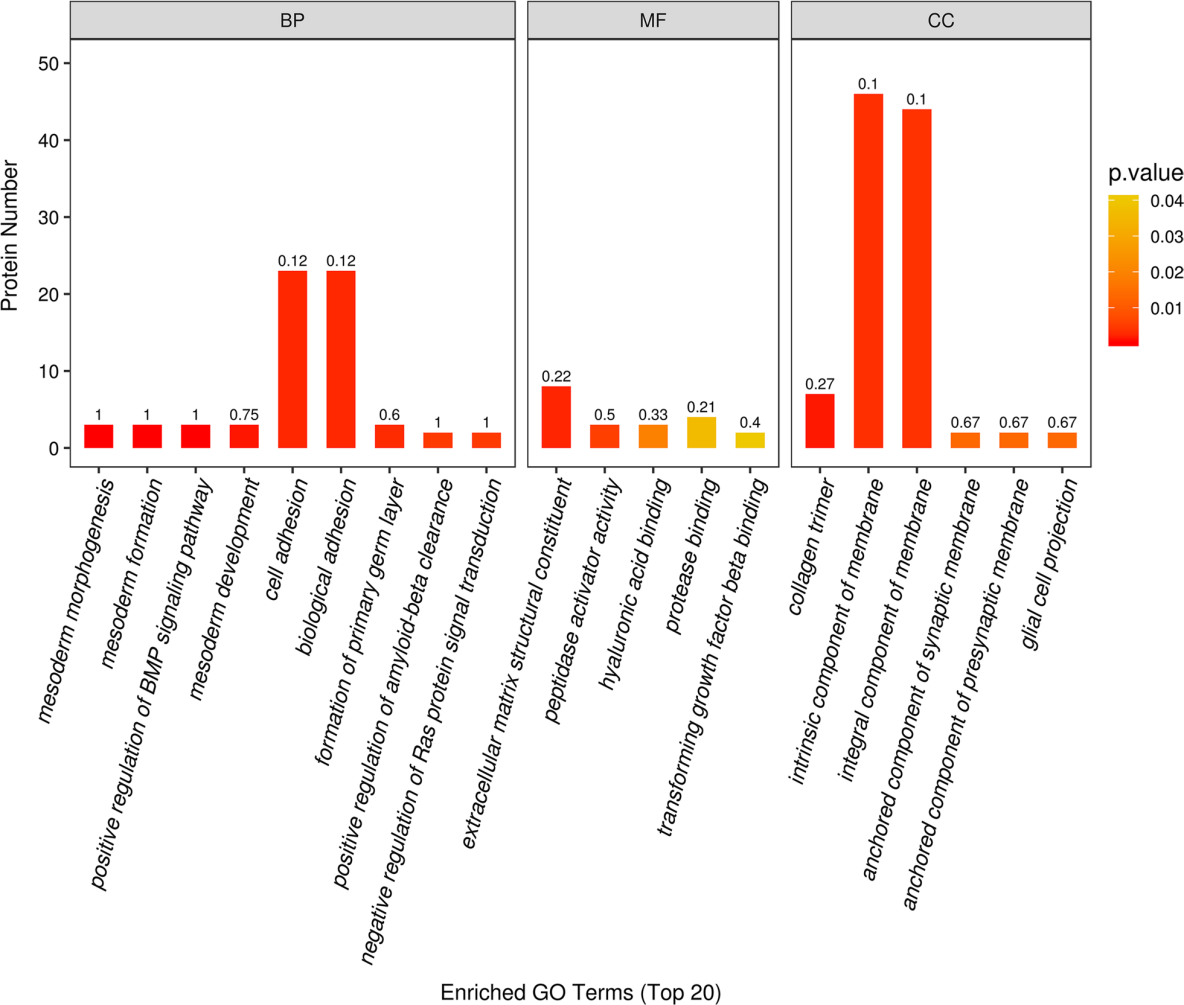
GO enrichment analysis of 125 differentially expressed proteins. The abscissa represents enriched GO function classifications, which are divided into three major categories: biological process (BP), molecular function (MF) and cellular component (CC). The ordinate represents the number of different proteins under each functional classification. The colour bars represent the significance of the enriched GO functional classification. The colour gradient represents the size of the *p* value (*p* < 0.05). The label at the top of the bar graph shows the enrichment factor (richFactor ≤ 1), which represents the ratio of the number of differentially expressed proteins annotated to a certain GO functional category to the number of identified proteins annotated to that GO functional category

We performed GO analysis to enrich and cluster the 125 differentially expressed proteins. The detailed information of the molecular functions, cellular components and biological processes is shown in Additional file 5. It was found that most of the proteins were involved in cell adhesion (n = 23), which as the main biological process identified by GO enrichment analysis (p < 0.05). In addition, GO annotation analysis revealed that the proteins were mainly distributed in the intrinsic components of the membrane (n = 46) and were mainly associated with the extracellular matrix (n = 8) (Fig. 2).

### KEGG pathway analysis

**Fig. 3:**
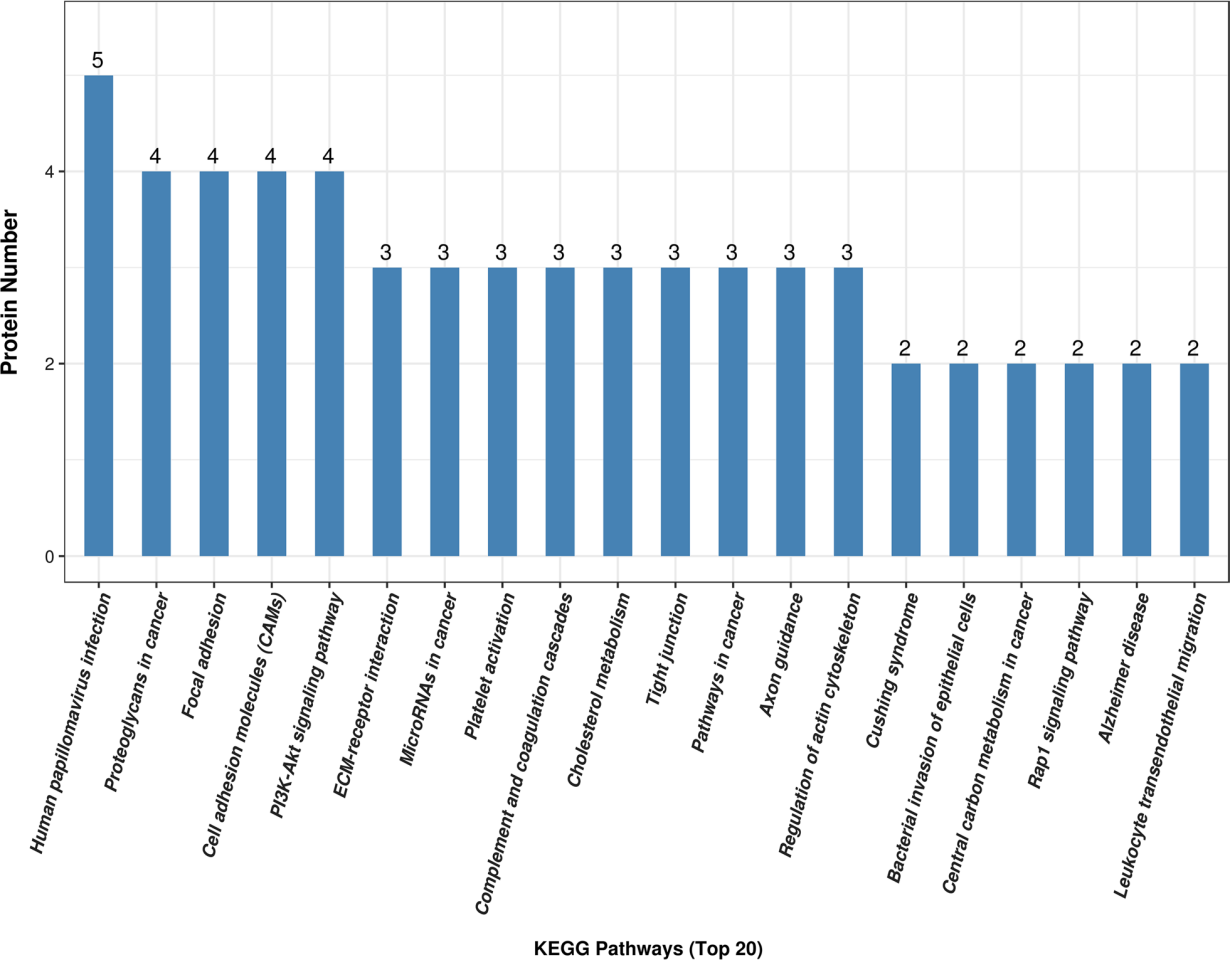
Top 20 enriched pathways according to KEGG functional classification analysis of 125 differentially expressed proteins. The ordinate represents the number of differentially expressed proteins contained in each KEGG pathway. The x-coordinate represents the significantly enriched KEGG pathway

**Fig. 4:**
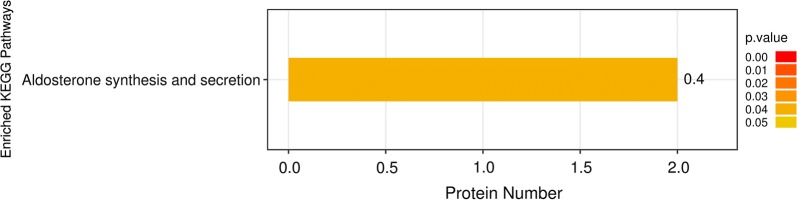
KEGG pathway enrichment analysis of 125 differentially expressed proteins. The ordinate represents the significantly enriched KEGG pathway. The x-coordinate represents the number of differentially expressed proteins contained in each KEGG pathway. The colour bars represent the significance of the enriched KEGG pathways. The colour gradient represents the size of *p* values (*p* < 0.05). The label at the top of the bar graph shows the enrichment factor (richFactor ≤ 1), which represents the proportion of the number of differentially expressed proteins involved in a KEGG pathway among all identified proteins

We next used KEGG analysis to search for enriched pathways including the 125 differential proteins. The detailed information of the KEGG pathway enrichment is shown in Additional file 6. According to the KEGG function classification analysis, focal adhesion, cell adhesion molecules, the phosphatidylinositide 3-kinase-protein kinase B (PI3K-Akt) signalling pathway, extracellular matrix (ECM)-receptor interactions, platelet activation, complement and coagulation cascades, cholesterol metabolism, and regulation of the actin cytoskeleton were among the top 20 enriched pathways (Fig. 3). The aldosterone synthesis and secretion pathway (p < 0.05) was determined to be significantly enriched according to KEGG analysis (Fig. 4).

### Proteins interaction analysis

**Fig. 5:**
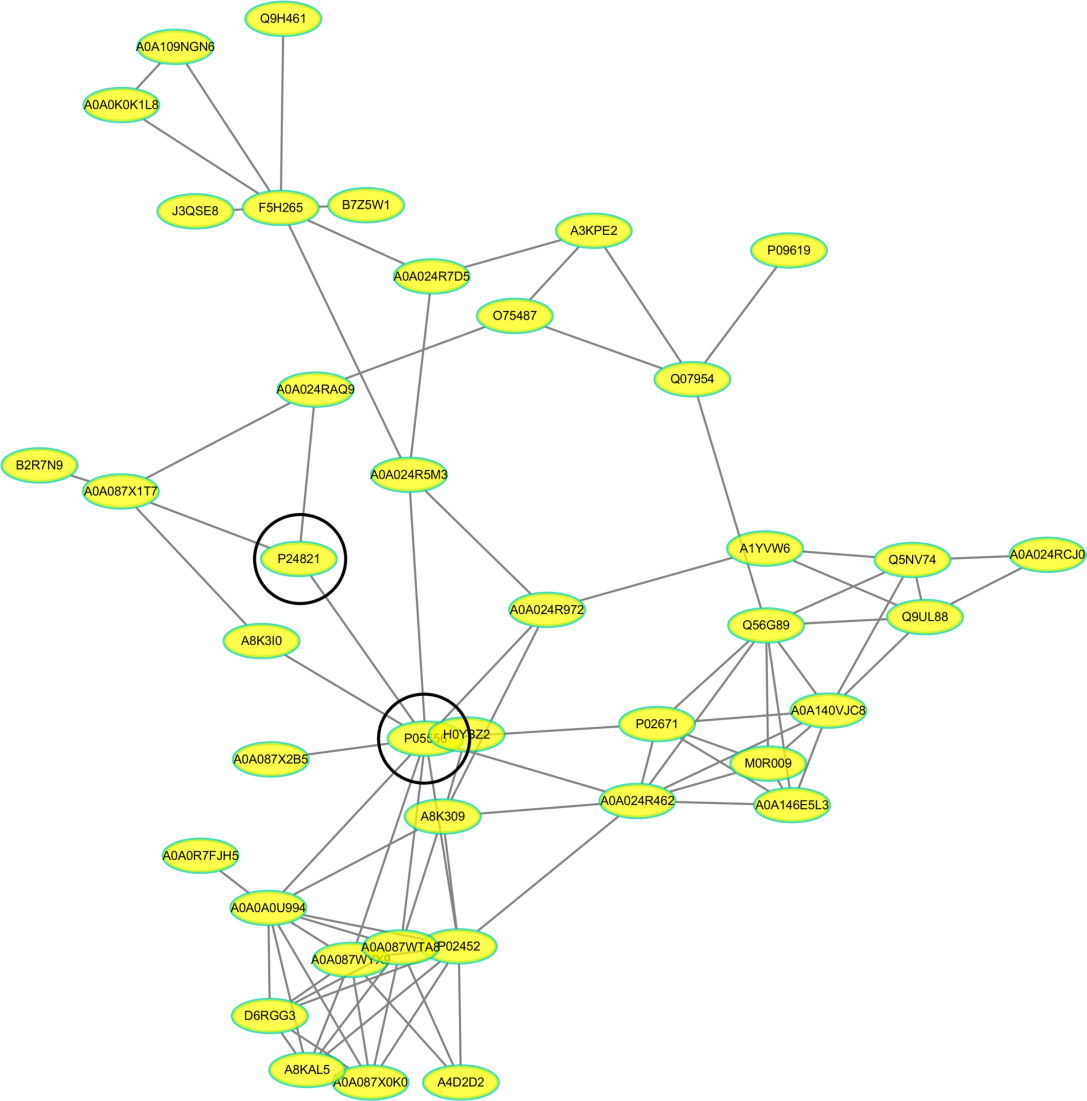
Differentially expressed protein interaction networks. In the protein interaction network, nodes represent proteins and lines represent protein–protein interactions. Yellow nodes are differentially expressed proteins. The number of proteins directly interacting with protein A is called the linkage degree of protein A. P24821 tenascin, P05556 integrin beta-1, A0A024RAQ9 versican, P02452 collagen alpha-1(I), P02671 Fibrinogen alpha, A0A024R462 fibronectin-1

A total of 41 proteins were predicted to have direct interactions among the 125 differentially expressed proteins according to PPI analysis. Interestingly, the core proteins (integrin beta-1 and tenascin) were involved in multiple pathways, such as focal adhesion, cell adhesion molecules, ECM-receptor interactions and the PI3K-Akt signalling pathway (Fig. 5). In addition, it was found from the network that these proteins were directly or indirectly related to collagen, fibronectin, and fibrinogen. Therefore, integrin beta-1 and tenascin were selected for further validation.

### ELISA analysis

**Fig. 6:**
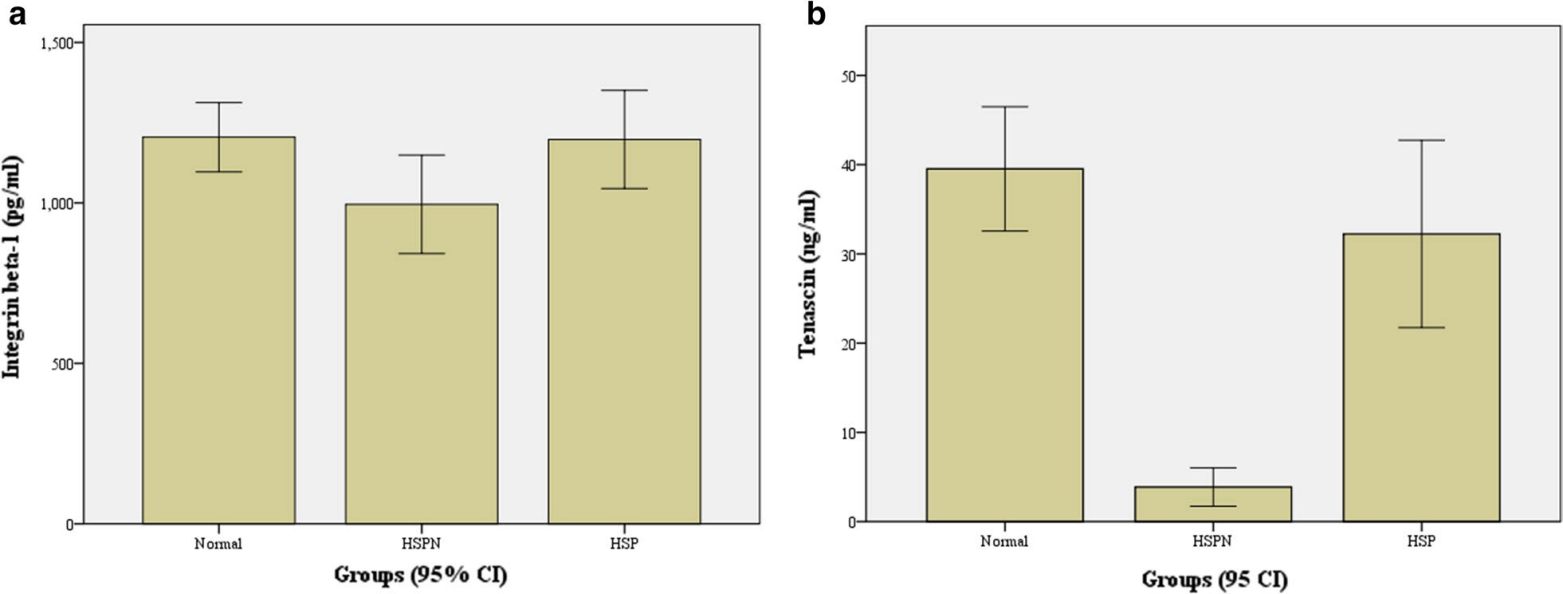
Urinary levels of integrin beta-1 and tenascin by ELISA in the validation cohort. **a** The urine concentration level of integrin beta-1 using ELISA kits. **b** The urine concentration level of tenascin using ELISA kits. The ordinate represents the concentration of proteins. The x-coordinate represents the group of validation. HSPN represents the group of Henoch-Schönlein purpura nephritis, Normal represents the healthy control group, and HSP represents the group of Henoch-Schönlein purpura

To validate the results of the proteomic analysis, two proteins (integrin beta-1 and tenascin) were chosen for further study, and their urinary levels in the validation cohort, including 30 HSPNs, 29 healthy controls, and 10 HSPs, were measured by using ELISA kits. The urinary levels results are shown in Fig. 6a, b. Integrin beta-1 and tenascin were significantly downregulated in HSPN patients compared to healthy controls (*p* < 0.05), but compared with HSP, integrin beta-1 had no significant difference (*p* = 0.508), while tenascin had a significant difference (*p* = 0.005) in HSPN.

## Discussion

Currently, renal involvement is critical for the prognosis of children with HSPN. Most of the need lies in the detection of HSPN patients at risk of occurrence and the progression of renal disease, and early diagnosis and treatment may greatly contribute to enhancing the survival of children. Monitoring changes in the urinary proteome may have an advantage in the diagnosis of kidney disease. The review of Julian BA et al. concluded urinary detection is more sensitive than current standard clinical testing and far less risky than renal biopsy, and so provides a useful tool for the diagnosis and monitoring of patients with IgA associated renal diseases [17]. Targeted proteomics is a hot research topic that can identify promising biomarkers of disease activity and organ involvement [18]. However, proteomic analysis of HSPN has rarely been studied, especially in urine. He XL et al. performed a comparative analysis of serum proteomes by LC‑MS/MS that revealed proteomic alterations in HSPN patients [19]. An analysis of urinary polypeptides from HSPN patients by electrophoretic methods detected urine proteins such as collagen alpha-1 (I) and (III) and alpha-1-antitrypsin [20]. To the best of our knowledge, no proteomic analysis of urine has been reported for the discovery of biomarkers and/or to describe the involvement of related biological pathways in children with HSPN. In the present study, we performed a comparative analysis of the urinary proteome to obtain insights into the pathogenesis in HSPN children using the DIA method via LC‑MS/MS. As a result, we found that 125 proteins were differentially expressed in urine form HSPN patients compared to that from healthy controls. Forty-one proteins were predicted to directly interact with each other.

According to GO annotation, our study found that most of the differentially expressed proteins are involved in cell adhesion. Cell adhesion linking cells to the ECM is crucial for maintaining the mechanical integrity of podocytes, which is a key component of the glomerular filtration barrier [21], and integrin beta-1 is the major cell–matrix adhesion receptor in podocytes, which connects laminin in GBM through various adaptor proteins to the intracellular actin cytoskeleton [22]. This process may be critical for the development of HSPN in children. According to KEGG function classification analysis, a variety of enriched pathways are involved in HSPN, such as focal adhesion, cell adhesion molecules, the PI3K-Akt signalling pathway, ECM-receptor interactions and regulation of the actin cytoskeleton, which include many common differentially expressed proteins (integrin beta-1, tenascin, collagen alpha-1 (I) and platelet-derived growth factor receptor) that participate in mesangial cell proliferation and fibrosis processes [23–25]. HSPN is driven by mesangial IgA deposits and the resulting mesangial cell proliferation and glomerular segmental sclerosis according to our renal biopsy results. The above-mentioned enriched pathways may account in part for the pathological changes associated with HSPN. In addition, our data showed that complement and coagulation cascades and platelet activation are predominantly involved in the pathway analysis. Several studies have reported that the activation of platelets and coagulation-complement crosstalk may be associated with a higher risk of thrombotic events [26]. HSPN is the pathological type of nephrotic syndrome associated with the highest incidence of thromboembolic events. Similarly, changes occur in aldosterone synthesis and secretion and cholesterol metabolism.

To validate the proteomic analysis results, the downregulation of the two core proteins by ELISA (integrin beta-1 and tenascin) was found to be consistent with the mass spectrometry results. However, the urine secretion level of integrin beta-1 in children with HSPN was similar to that of children with HSP, which may be related to the participation of integrin beta-1 in multiple pathways in disease. However, the urine secretion level of tenascin was significantly different among HSPN children and HSP children. Therefore, tenascin might be a novel HSPN-related biomarker that can be used to diagnose early renal damage in children with HSPN. Tenascin, a large oligomeric glycoprotein, is a ubiquitous extracellular matrix protein of human renal tissue under normal and pathological conditions [27]. Tenascin is expressed on the cell surface and plays a critical role in a wide array of biological processes, including the immune response, inflammation, and morphogenesis. Tenascin rapidly responds to inflammation and injury, and urine excretion of tenascin may reduce the fibrogenic response to acute tissue damage. Studies have identified tenascin as a major constituent of the fibrogenic niche that promotes fibroblast proliferation [28]. Ozkan H et al. indicated tenascin as a potential urine biomarker for acute kidney injury using comparative proteomics [29]. In addition, expression of the tenascin protein may be an indicator of disease chronicity, and mRNA expression of tenascin may be an indicator of disease activity with renal involvement [30]. Thus, it may be worthwhile to further investigate the predictive role of urine tenascin on the progression to HSPN.

This study has several limitations. We only performed LC–MS/MS on a few urine samples, and we only outlined the possible candidate proteins involved. It is necessary to increase the accuracy and reduce the variability of our analyses through further verification in a large sample size. We did not assess other glomerular diseases using biological analysis.

## Conclusions

In summary, our study suggests that 125 differentially expressed proteins and multiple enrichment pathways are associated with the pathogenesis of HSPN in children using the DIA method via LC–MS/MS. The value of urinary proteomics in disease recognition was further confirmed. The discovery of differential proteins lays the foundation for searching for potential biomarkers of HSPN in children.

## Supplementary information

**Additional file 1.** Clinical characteristics of patients in group A.

**Additional file 2.** Clinical characteristics of patients in group B.

**Additional file 3.** Clinical characteristics of patients in group C.

**Additional file 4.** Raw data of the information of 125 proteins.

**Additional file 5.** The detail information of gene ontology (GO) enrichment analysis.

**Additional file 6.** The detail information of Kyoto Encyclopedia of Genes and Genomes (KEGG) pathway enrichment analysis.

## Data Availability

All data generated or analysed during this study are included in this published article and its additional files.

## Abbreviations

HSPN: Henoch-Schönlein purpura nephritis
HSP: Henoch-Schönlein purpura
IgAN: IgA nephropathy
LC–MS/MS: Liquid chromatography-tandem mass spectrometry
DIA: Data independent acquisition
DDA: Data dependent acquisition
GO: Gene ontology
KEGG: Kyoto Encyclopedia of Genes and Genomes
KAAS: KEGG Automatic Annotation Server
ELISA: Enzyme-linked immunosorbent assay
ECM: Extracellular matrix
PI3K-Akt: Phosphatidylinositide 3-kinase-protein kinase B
FDR: False discovery rate
PPI: Protein–protein interaction
OD: Optical density
HRP: Horseradish peroxidase
